# The Combined Effects of Short-Term Exposure to Multiple Meteorological Factors on Unintentional Drowning Mortality: Large Case-Crossover Study

**DOI:** 10.2196/46792

**Published:** 2023-07-20

**Authors:** Yingyin Liu, Xiaomei Dong, Zhixing Li, Sui Zhu, Ziqiang Lin, Guanhao He, Weiwei Gong, Jianxiong Hu, Zhulin Hou, Ruilin Meng, Chunliang Zhou, Min Yu, Biao Huang, Lifeng Lin, Jianpeng Xiao, Jieming Zhong, Donghui Jin, Yiqing Xu, Lingshuang Lv, Cunrui Huang, Tao Liu, Wenjun Ma

**Affiliations:** 1 Department of Public Health and Preventive Medicine School of Medicine Jinan University Guangzhou China; 2 Department of Nosocomial Infection Management Affiliated Nanfang Hospital of Southern Medical University Guangzhou China; 3 Zhejiang Provincial Center for Disease Control and Prevention Hangzhou China; 4 Guangdong Provincial Institute of Public Health Guangdong Provincial Center for Disease Control and Prevention Guangzhou China; 5 Yunnan Provincial Center for Disease Control and Prevention Kunming China; 6 Jilin Provincial Center for Disease Control and Prevention Changchun China; 7 Guangdong Provincial Center for Disease Control and Prevention Guangzhou China; 8 Hunan Provincial Center for Disease Control and Prevention Changsha China; 9 Vanke School of Public Health Tsinghua University Beijing China; 10 Disease Control and Prevention Institute of Jinan University School of Medicine Jinan University Guangzhou China

**Keywords:** drowning, exposure mixture, quantile g-computation, environmental epidemiology, meteorological factor

## Abstract

**Background:**

Drowning is a serious public health problem worldwide. Previous epidemiological studies on the association between meteorological factors and drowning mainly focused on individual weather factors, and the combined effect of mixed exposure to multiple meteorological factors on drowning is unclear.

**Objective:**

We aimed to investigate the combined effects of multiple meteorological factors on unintentional drowning mortality in China and to identify the important meteorological factors contributing to drowning mortality.

**Methods:**

Unintentional drowning death data (based on International Classification of Diseases, 10th Edition, codes W65-74) from January 1, 2013, to December 31, 2018, were collected from the Disease Surveillance Points System for Guangdong, Hunan, Zhejiang, Yunnan, and Jilin Provinces, China. Daily meteorological data, including daily mean temperature, relative humidity, sunlight duration, and rainfall in the same period were obtained from the Chinese Academy of Meteorological Science Data Center. We constructed a time-stratified case-crossover design and applied a generalized additive model to examine the effect of individual weather factors on drowning mortality, and then used quantile g-computation to estimate the joint effect of the mixed exposure to meteorological factors.

**Results:**

A total of 46,179 drowning deaths were reported in the 5 provinces in China from 2013 to 2018. In an effect analysis of individual exposure, we observed a positive effect for sunlight duration, a negative effect for relative humidity, and U-shaped associations for temperature and rainfall with drowning mortality. In a joint effect analysis of the above 4 meteorological factors, a 2.99% (95% CI 0.26%-5.80%) increase in drowning mortality was observed per quartile rise in exposure mixture. For the total population, sunlight duration was the most important weather factor for drowning mortality, with a 93.1% positive contribution to the overall effects, while rainfall was mainly a negative factor for drowning deaths (90.5%) and temperature and relative humidity contributed 6.9% and –9.5% to the overall effects, respectively.

**Conclusions:**

This study found that mixed exposure to temperature, relative humidity, sunlight duration, and rainfall was positively associated with drowning mortality and that sunlight duration, rather than temperature, may be the most important meteorological factor for drowning mortality. These findings imply that it is necessary to incorporate sunshine hours and temperature into early warning systems for drowning prevention in the future.

## Introduction

Drowning is the process of experiencing respiratory impairment from submersion or immersion in liquid [1]; it is a serious public health problem worldwide. The World Health Organization (WHO) reported that an estimated 236,000 people died from drowning in 2019. Globally, drowning is the third leading cause of death by unintentional injury, accounting for 7% of all injury-related deaths [2]. Despite the downward trend in drowning mortality rates [3], there are still considerable disparities across countries. Developed countries tend to have lower drowning mortality; for example, in the United States, the drowning mortality rate was 1.23 per 100,000 from 2010 to 2019 [4]. However, developing regions, like Southeast Asia, are the regions with the highest drowning mortality rates in the world. In the last decades, China has made great progress in drowning prevention and control, and the drowning mortality rate has decreased from 15.09 per 100,000 in 1990 to 3.97 per 100,000 in 2019 [5]; however, this is still a high drowning burden compared with most other countries.

Prior studies on drowning mainly focused on epidemiological characteristics or risk factor analysis [6-8], clinical first aid [9,10], and intervention policy assessment [11,12]. Few studies have investigated the association of meteorological factors with drowning. Among these few studies, several reports indicate that there is a clear seasonal distribution of drowning deaths, with the highest drowning mortality in the summer [13]. The summer heat itself may not increase the risk of drowning, but it may lead to changes in behavior that increase susceptibility to drowning, such as increased water-related activities, increased alcohol consumption, or decreased use of personal flotation devices [14]. In terms of humidity, several studies report that high relative humidity (RH) at high temperatures significantly increases body temperature and increases the chance that people engage in water-related activities such as swimming [15], which in turn leads to an increased risk of drowning. Flooding due to extremely heavy rainfall is also an important cause of drowning mortality. For example, in July 2021, a flood occurred in Henan Province, China, due to persistent extremely heavy rainfall, and this incident resulted in hundreds of drowning deaths [16]. Even though there is no research on the association between duration of sunlight (DS) and drowning, there is research illustrating the importance of daylight hours for those who plan to enjoy swimming in a lake or outdoor pool, thereby potentially increasing the chance of drowning [17]. These studies focused on the effects of single weather factors on drowning. However, in the real world, people are exposed to multiple weather factors simultaneously, not only one at a time. Regrettably, no prior studies have examined the combined effects of exposure to multiple meteorological factors on drowning.

Indeed, it is a challenge to quantify the combined effects of multiple meteorological exposure factors on drowning using traditional statistical models. In order to reduce collinearity in the model, previous multivariate regression models, such as linear regression, generalized linear regression, and generalized additive models (GAMs) have usually explored the association between a single or limited environmental factors and health outcomes. However, this approach could not accurately reveal the true impact of multiple environmental exposures [18]. In recent years, several advanced statistical methods, such as quantile g-computation, have been developed to examine the joint health effects of exposure mixture [19], which provides an opportunity for combined-effect analysis of exposure mixture in environmental epidemiology. Quantile g-computation not only allows for nonlinear correlation analysis, but also calculates the positive and negative weights contributed by each exposure.

This study aimed to investigate the combined effects of multiple meteorological factors, including mean temperature (MT), RH, DS, and rainfall, on drowning mortality based on a data set from 5 provinces in China using a quantile g-computation model [19] and to further quantify the relative importance of each meteorological factor. Our findings may provide scientific information for developing an early warning system for drowning based on multiple meteorological factors to prevent drowning in the context of global warming.

## Methods

### Data Collection

In this study, the study sites in China were Guangdong Province in south China, Hunan Province in the middle part of the Yangtze River, Zhejiang Province on the southeast coast of China, Yunnan Province in southwest China, and Jilin Province in northeast China. Guangdong and Hunan are both regions with rich sunlight, heat, and water resources. Zhejiang Province is rich in marine resources. Both Jilin and Yunnan provinces have many rivers and lakes.

**Figure 1:**
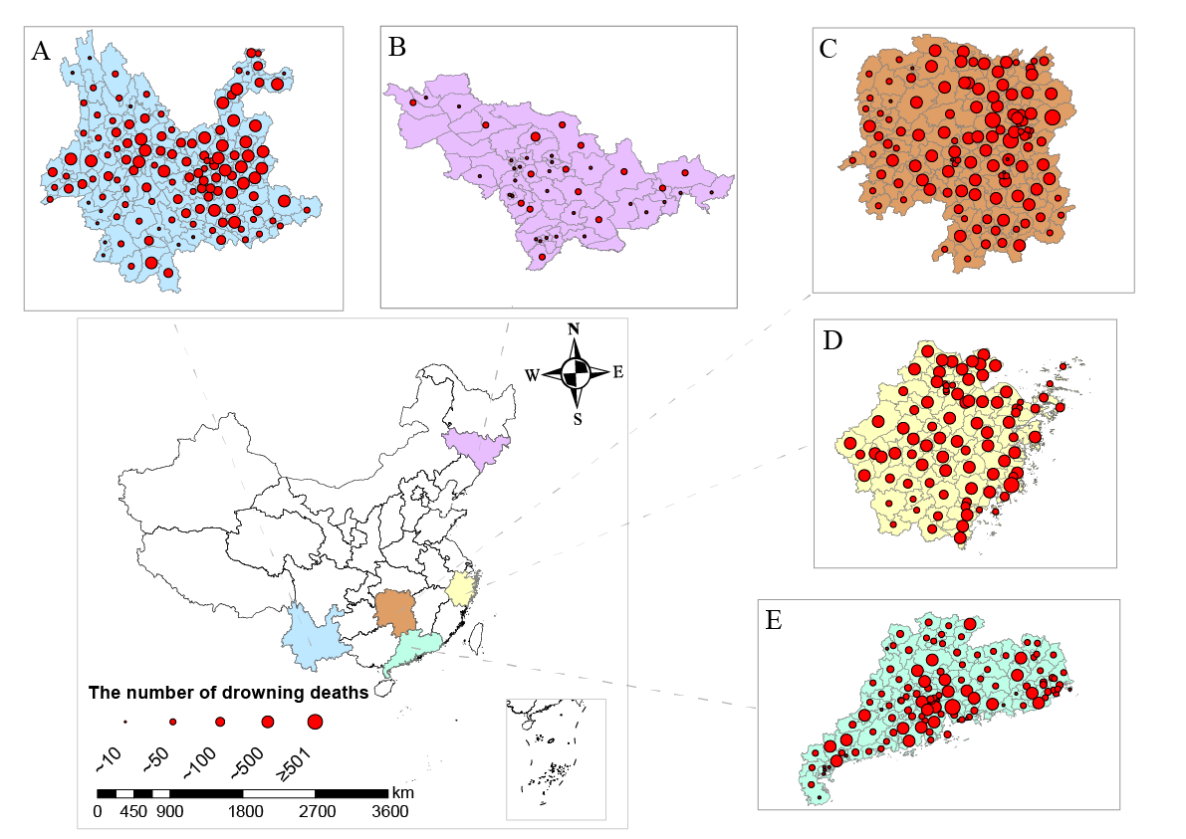
Spatial distribution of drowning deaths at the district and county level in 5 provinces. (A) Yunnan Province; (B) Jilin Province; (C) Hunan Province; (D) Zhejiang Province; (E) Guangdong Province.

Drowning mortality data from January 1, 2013, to December 31, 2018, in these 5 Chinese provinces were collected from the Provincial Disease Surveillance Points System. The information on each death included the date of death, cause of death, address, gender, age, education level, and cause of death code. In the International Classification of Diseases, 10th Edition (ICD-10), the codes W65 to 74 are unintentional drowning deaths, excluding drownings due to cataclysms or transportation accidents. The spatial distribution of drowning deaths in the 5 provinces is shown in Figure 1.

Daily meteorological data, including daily MT, RH, DS, and rainfall from 2013 to 2018 were collected from the China Meteorological Science Data Center, which collects data from 698 meteorological surveillance points across the country [20]. In order to obtain accurate daily MT and RH at a county level, we used Australian National University Splines (ANUSPLIN) thin plate smoothing [21] to interpolate station meteorological data to form raster data for daily MT and RH with a resolution of 0.01° × 0.01°. In the spatial interpolation model, we introduced latitude, longitude, and altitude as independent variables. The interpolation was validated using the 10-fold crossover method with good prediction accuracy, as previously described [22]. Daily mean DS and rainfall amount were calculated by averaging the values of all stations in each city because these 2 weather factors returned inaccurate data from interpolation in our preliminary analysis.

Air pollution might be a confounding factor, so we also collected data on fine particulate matter (PM2.5) as a representative of air pollutants to control for possible confounding effects. Daily air pollution data (ie, PM2.5) were derived from the National Urban Air Quality Real-Time Release Platform [23]. Because the air pollution surveillance points did not completely cover our study provinces, the land use regression method, based on the random forest model, was used to predict ambient air pollutants; the model showed good prediction capability [24].

### Ethics Approval

Ethical approval was obtained from the Medical Research Ethics Committee of Guangdong Provincial Center for Disease Control and Prevention (2019025), and all procedures followed the ethical standards specified by the institution.

### Statistical Analysis

This study used a case-crossover design based on temporal stratification, which was further adapted from the case-crossover design of Levy et al [25]. The basic principle is to stratify time so that each case with an outcome is treated as a case group, while exposures in the same year, month, and day of the week are treated as controls, meaning that each case has 3 to 4 controls, thereby effectively controlling for confounding factors, including long-term trends, gender, and age, and effectively avoiding control selection bias in the original case-crossover design and enabling unbiased estimates to be obtained. This research design has been widely used to investigate the effects of short-term environmental exposure on population health, especially in the field of health-effect assessment of meteorological factors [26] and air pollution [27].

Descriptive analyses were represented by numbers, means, maximum values, minimum values, and medians (quartiles), respectively. Spearman correlation was used to examine the correlations between meteorological factors and PM2.5.

Few previous studies have reported the effects of wind speed and air pressure on drowning. Moreover, after preliminary analysis, we observed that wind speed and air pressure had little effect on drowning mortality (Multimedia Appendix 1). Therefore, these 2 weather factors were not included in the joint-effect analysis. In our study, we first conducted individual meteorological factor analysis using a GAM [28] after adjusting for confounding factors (ie, PM2.5) to assess the individual effect of each meteorological factor (MT, RH, DS, and rainfall) on drowning mortality. The GAM equation is as follows:

Logit(case)=s(Variable,df)+s(PM_2.5_,df)+α,

where *Logit()* is the connection function, *case* indicates the case type with 1 for cases and 0 for controls; *Variable* represents the meteorological factors to be analyzed, that is, MT, RH, DS and rainfall; *PM2.5* is the concentration of PM2.5; *s()* is the nonparametric smoothing function, which is used to fit the nonlinear relationship between meteorological factors (ie, PM2.5 and drowning, respectively), with 3 degrees of freedom selected by generalized cross-validation [29]; and *α* denotes the intercept term.

Second, in order to further estimate the joint effect of multiple meteorological factor exposure on drowning mortality, we used a quantile g-computation model [30]. Quantile g-computation is a new approach to estimate the joint effects of exposure mixture [19]. It has been used to evaluate the change in health outcome for each quantile of mixed exposure to multiple environmental factors [31-33], and it does not require the same effect directions between exposure variables and outcome [18]. In this study, the model was implemented through categorizing MT, RH, DS, and rainfall in quartiles, 
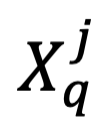
, coded as 0, 1, 2, and 3. The mixture is first fitted nonlinearly to drowning and then linearized according to the results. The quantile g-computation model equation is as follows (confounders *Z* could also be included):




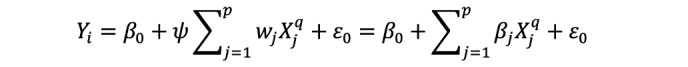




where 
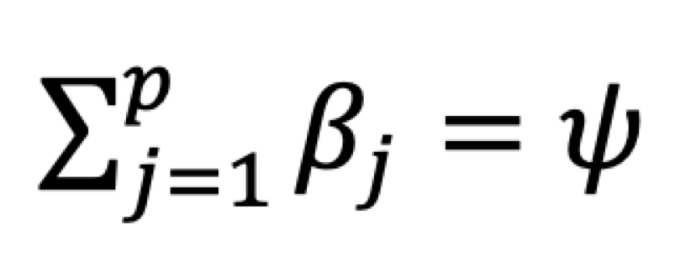
 (the change in drowning for a 1-unit change in all exposures) and each meteorological factor is given a negative or positive weight. The quantile g-computation estimator of the exposure response, *ψ*, is the sum of the regression coefficients across the included exposures. If all *β_j_* are in the same direction, then the weight for each component (indexed by *k*) is defined as 
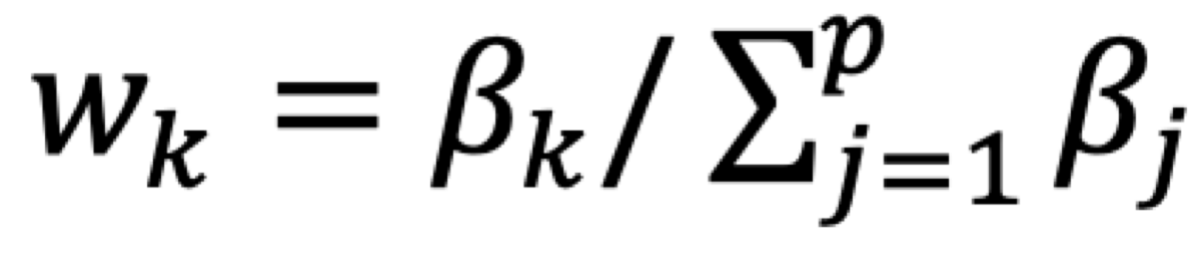
, which is the proportion of the effect due to that component and sums to 1.0. If factors have different directions of effect, then the weights are interpreted as the proportion of the positive or negative partial effect, and the positive and negative weights together sum to 2.0 [32]. After obtaining regression coefficients for 71 cities, values from different cities were combined using meta-analysis to obtain the exposure-response relationship between exposure mixture and drowning risk. The results of the nonlinear fit were expressed as exposure response curves.

The linearized results were expressed in terms of excess risk (ER) with the 95% CI. The formula for ER is as follows:

ER = [exp(β) – 1] × 100%

where *β* is the effect of exposure mixture in meta-analysis.

Moreover, we used the quantile g-computation model to conduct subgroup analysis by sex (male, female), age group (<25 years, 25-64 years, ≥65 years) and season (the cold season, from October to March, and the warm season, from April to September). The following formula was used for the between-group variability test:




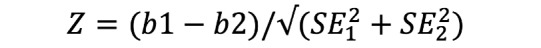




where *b_1_* and *b_2_* are the effect estimates of the 2 groups, respectively, and *SE_1_* and *SE_2_* are the SEs of the estimated effects of the 2 groups, respectively [34].

Sensitivity analysis was performed by changing the parameters of the model as well as by adjusting the confounding factors in order to verify the stability and reliability of the model. In the GAM, we conducted a sensitivity analysis by changing the degrees of freedom (*df*=2,4,5) for MT, RH, DS, and rainfall. In the quantile g-computation model, model 1 removed the confounding factor PM2.5 for sensitivity analysis. Model 2, model 3, and model 4 used 2-day, 3-day, and 4-day moving averages for MT, RH, and PM2.5 for sensitivity analysis.

All tests were conducted 2-sided, and effects with *P*<.05 were considered statistically significant. All analyses were conducted with R (version 4.1.0; R Foundation for Statistical Computing) and the *mgcv*, *qgcomp*, *mvmeta*, and *metafor* R packages.

## Results

### Descriptive Analysis

There were 46,179 drowning deaths in the 5 Chinese provinces from 2013 to 2018, with an average of 21 and a maximum of 68 drowning deaths per day. Each city had an average of 1 drowning death per day, with a maximum of 10 deaths (Table 1). A total of 17,408 people died from drowning during the summer seasons. The number of drowning deaths was the highest in Hunan Province and the lowest in Jilin Province. The number of drowning deaths was much higher for males (n=29,805, 64.5%) than females (n=16,374, 35.5%). The daily MT, RH, rainfall and PM2.5 during the study period were 20.4 °C, 76.7%, 4.5 mm, and 37.6 µg/m^3^, respectively (Table 2).

**Table 1:** Descriptive statistics of meteorological factors and daily drowning deaths by season, province, sex, and age group from 2013 to 2018.

Characteristics			Drowning deaths overall (n=46,179), n (%)		Drowning deaths per day, n										
					Mean (total=21.1)		Minimum (total=3)		P_25_^a^ (total=14)		P_50_^b^ (total=19)		P_75_^c^ (total=26)		Maximum (total=68)
In each city			N/A^d^		1.3		1		1		1		2		10
**Season**															
	Warm	29,145 (63.1)		26.5		6		20		26		32		68	
	Cold	17,034 (36.9)		15.6		3		12		15		19		40	
**Sex**															
	Male	29,805 (64.5)		13.6		1		8		12		18		49	
	Female	16,374 (35.5)		7.5		1		5		7		10		25	
**Age (years)**															
	≤24	13,827 (30)		6.4		1		3		5		9		26	
	25-64	16,918 (36.6)		7.8		1		5		7		10		27	
	≥65	15,434 (33.4)		7.1		1		5		7		9		23	

^a^P_25_: 25th percentile.

^b^P_50_: 50th percentile.

^c^P_75_: 75th percentile.

^d^N/A: not applicable.

**Table 2:** Descriptive statistics of meteorological factors from 2013 to 2018.

Meteorological factors	Mean	Minimum	P_25_^a^	P_50_^b^	P_75_^c^	Maximum
Average temperature (°C)	20.6	0.1	15.4	21.8	27.1	33.0
Relative humidity (%)	76.9	50.6	70.6	77.9	84.2	95.2
Sunlight duration (hours)	5.3	0.0	1.3	5.6	8.8	13.9
Rainfall (mm)	2.9	0.0	0.0	0.2	2.7	35.0
PM2.5 (µg/m^3^)	38.1	3.6	20.7	31.5	47.6	302.9

^a^P_25_: 25th percentile.

^b^P_50_: 50th percentile.

^c^P_75_: 75th percentile.

### Individual Effect of Meteorological Factors on Drowning Mortality

**Figure 2:**
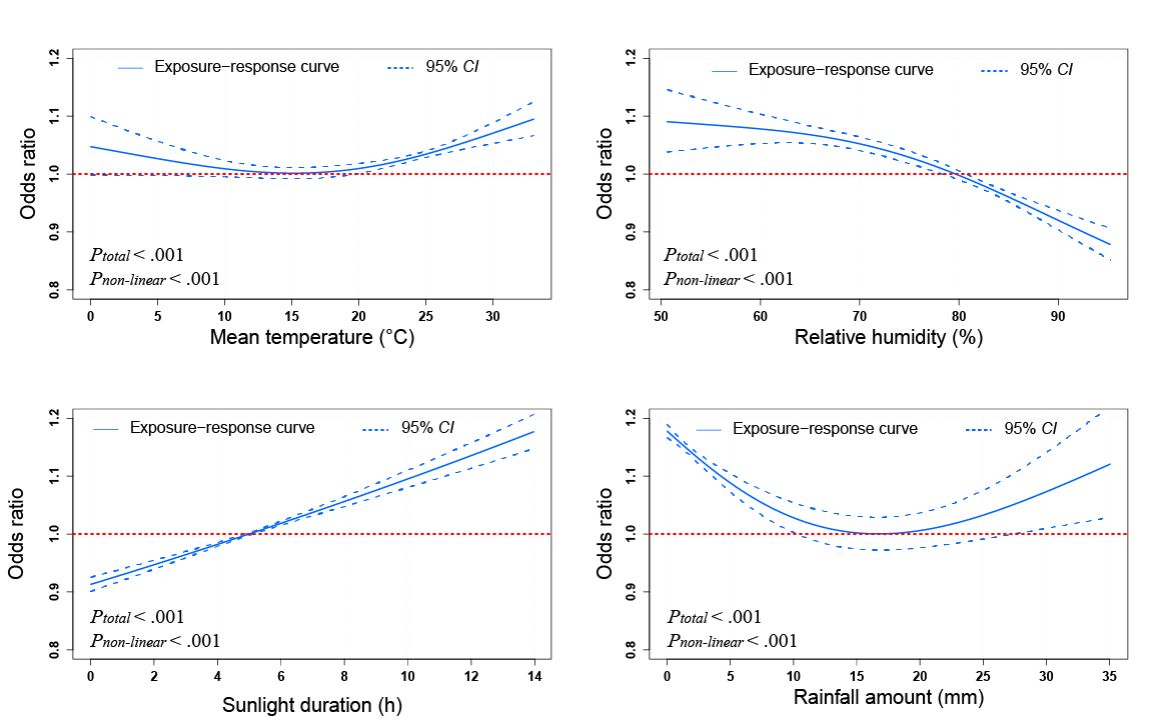
The associations of daily mean temperature, relative humidity, sunlight duration, and rainfall with drowning mortality in generalized additive models. Solid lines represent the exposure-response curve; dotted lines represent the 95% CI.

Among the 4 meteorological factors, the strongest correlation was observed between RH and DS (r=–0.61; *P*<.05; Multimedia Appendix 2). As shown in Figure 2, in an individual-effect analysis, there were U-shaped relationships between MT, rainfall and drowning mortality, with the lowest risk at 15.4 °C for MT and 16.6 mm for rainfall, respectively. RH was negatively associated with drowning mortality, while DS was positively associated with drowning mortality.

In the sensitivity analysis of the GAM, the relationship curves for MT, RH, DS, and rainfall with drowning deaths changed little after changing the degrees of freedom in the spline function. (Multimedia Appendix 3)

### Combined Effect of Multiple Meteorological Factors on Drowning Mortality

**Figure 3:**
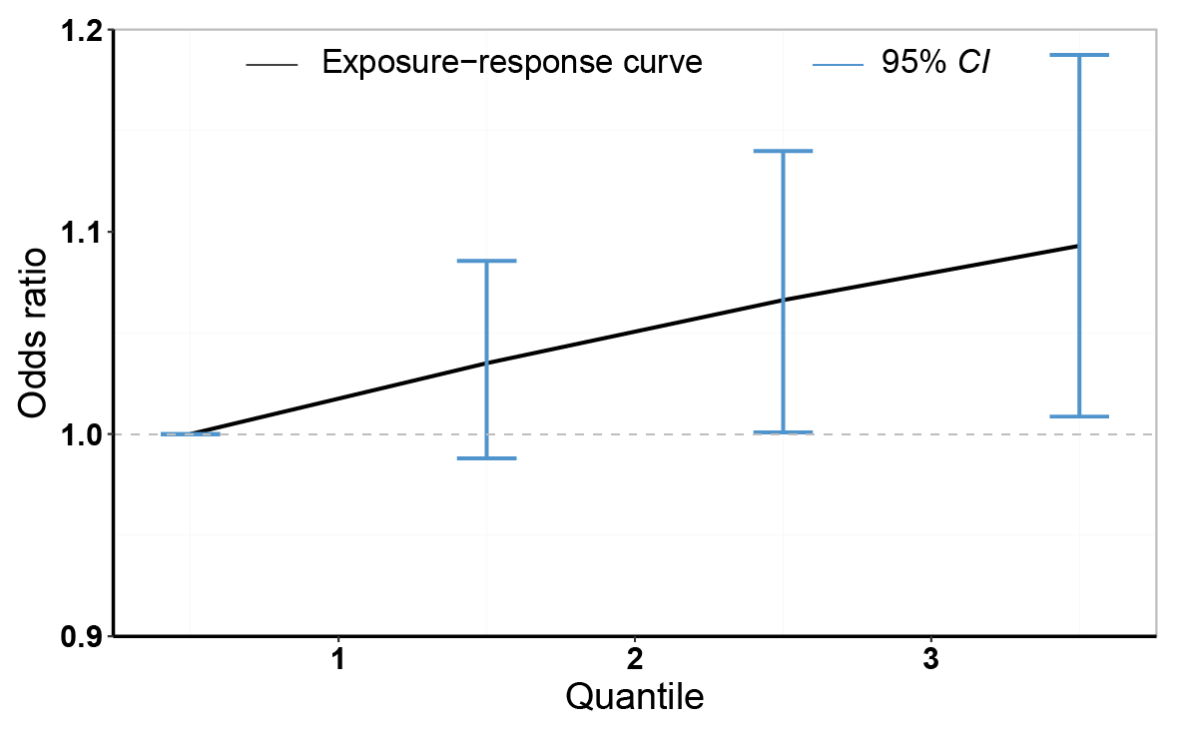
Exposure-response relationships between meteorological exposure mixture and drowning risk in 71 cities from a quantile g-computation analysis. The line is the exposure response curve, and the blue shaded area is the 95% CI at each percentile.

In the exposure mixture analysis using quantile g-computation, mixed exposure to the 4 meteorological factors was approximately linearly associated with the risk of drowning. The risk tended to increase with each percentile (Figure 3). Meanwhile, we conducted an effect modification analysis of city-level characteristics and found that the risk of drowning caused by meteorological exposure mixture was higher in cities with higher population density, while urban gross domestic product, latitude, longitude, and altitude were found to have only weak modification effects (Multimedia Appendix 4).

**Figure 4:**
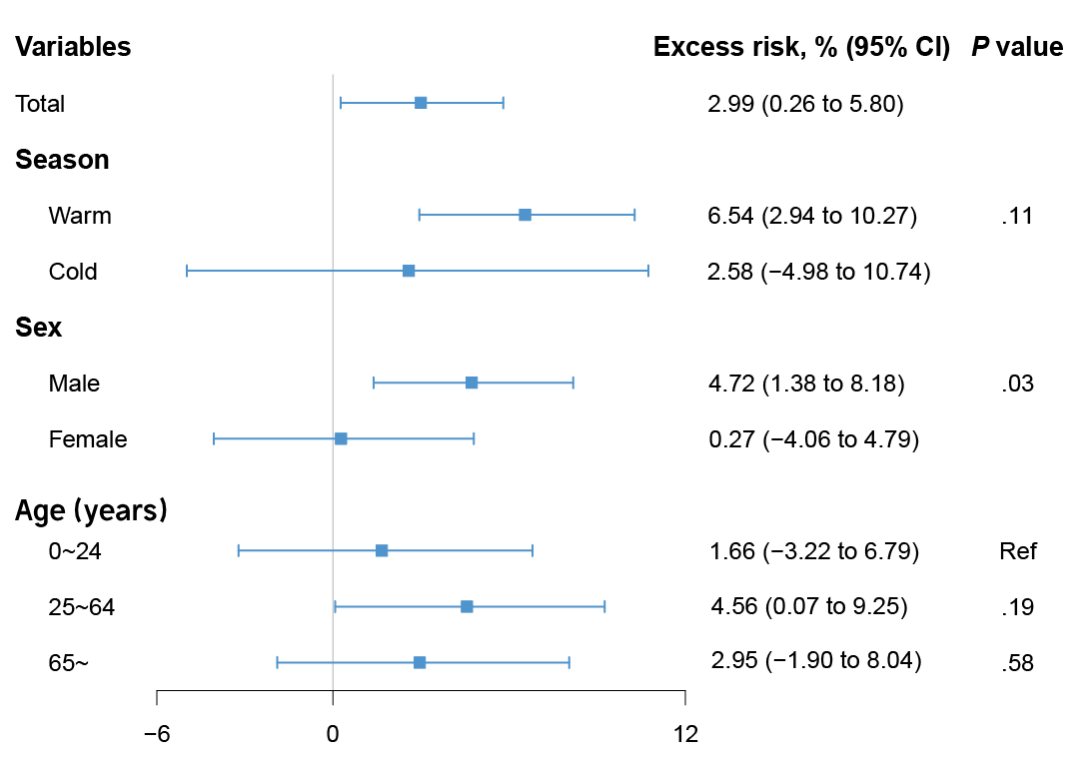
Excess risk (as a percentage) of drowning mortality per quartile increase in mixed daily mean temperature, relative humidity, sunlight duration, and rainfall in 71 cities.

After linearization, we observed a 2.99% (95% CI 0.26%-5.80%) drowning mortality increase per quartile rise in the 4 meteorological factors (Figure 4). Subgroup analyses showed that the effect for males was higher than for females with a statistically significant difference (*Z*=2.21; *P*=.03). The risk of drowning was heterogeneous across age groups in combined effects. Although those aged 25 to 64 years had the highest risk (ER 4.56%, 95% CI 0.07%-9.25% per quartile rise in the 4 meteorological factors), the difference among all age groups was not statistically significant. The drowning risk attributed to mixed exposure to the 4 weather factors in the warm season seemed higher than in the cold season, but the difference was also not statistically significant (*Z*=1.61; *P*=.11).

**Figure 5:**
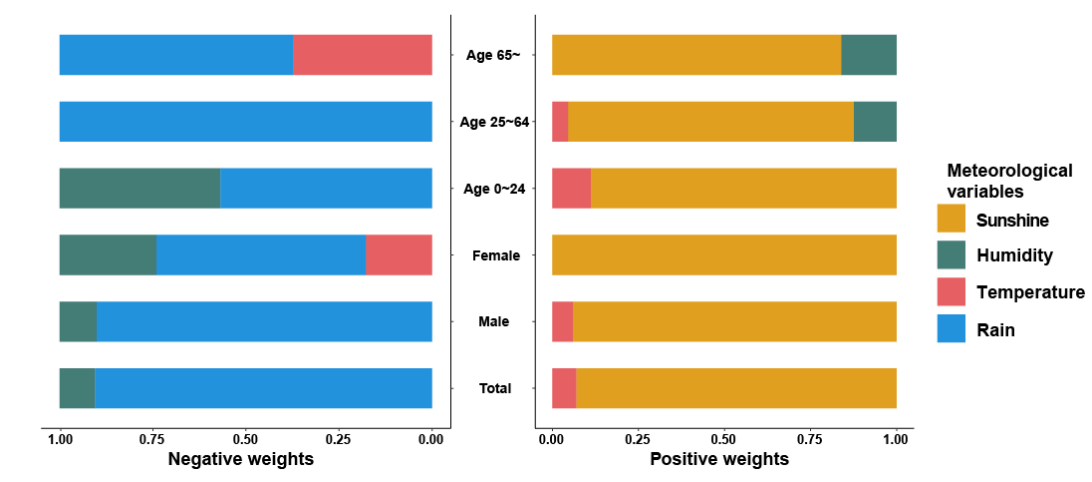
Weights representing the proportion of the positive or negative partial effect of each meteorological factor in quantile g-computation model by sex and age group.

Figure 5 further shows the relative contribution of each meteorological factor to the combined effect. Sunlight duration had a positive weight with a much higher proportion among all subgroups: 93.1%, 94.1%, 100%, 88.9%, 83.1%, and 83.9% for the total population, males, females, and people aged 0 to 24 years, 25 to 64 years, and ≥65 years, respectively. RH negatively contributed to the combined effect in the total population (9.5%), females (26%), males (9.9%), and young people aged 0 to 24 years (43.2%) and positively contributed in people aged 25 to 64 years (12.4%) and ≥65 years (16.1%). Temperature contributed positively in the total population (6.9%), males (5.9%), the population aged 0 to 24 years (11.1%), and the population aged 25 to 64 years (4.5%), while it made a negative contribution in females (17.8%) and people aged ≥65 years (37.2%). Rainfall always contributed negatively to drowning mortality among all populations (90.5%, 90.2%, 56.2%, 56.8%, 100%, and 62.8% for the total population, males, females, people aged 0 to 24 years, 25 to 64 years, and ≥65 years, respectively; Figure 5).

In a quantile g-computation model sensitivity analysis, the results were basically stable whether adjusting or not adjusting for PM2.5 in the model. The excess risk of drowning increased when moving averages of each weather factor were used in the model (Multimedia Appendix 5).

## Discussion

### Principal Findings

In this study, we examined the combined effect of daily MT, RH, DS, and rainfall on drowning mortality in China. We found that for every quartile increase in meteorological factors, the risk of drowning death in the population increased by 2.99%. We also observed that the 4 meteorological factors had a positively combined effect on drowning mortality in all subgroups except those aged 0 to 24 years; adults aged 25 to 64 years may be more susceptible to mixed exposure to the 4 meteorological factors. Moreover, we showed that DS, rather than MT, was the most important meteorological factor contributing to drowning mortality. These findings have important public health implications for developing an early warning system to reduce drowning mortality in the context of global warming.

### Results Interpretation and Implications

An increasing number of epidemiological studies have examined the relationship between exposure to single meteorological factors and the risk of drowning. Consistent with our findings, a study conducted by Chauvin et al [35] reported an increase in the risk of drowning death as ambient temperature rose. When the human body is exposed to high temperatures, the blood vessels in the skin dilate rapidly and sweating increases to accelerate the discharge of excess heat; when entering the water, heavy sweating could lead to electrolyte deficiencies, and swimming in water at this time may lead to dangerous reactions, such as convulsions [36]. Therefore, high temperatures increase the risk of drowning. Additionally, alcohol consumption at high temperatures is an important risk factor for drowning [37,38], as this could affect physiological reactions and behavioral patterns, thereby increasing the risk of drowning. A prior study reported that alcohol consumption was positively correlated with MT [39]. Individuals that consume alcohol have an increased risk of drowning due to greater skin blood flow and sweating. Also, alcohol can cross the human cerebrovascular barrier directly and affect mental and cognitive performance, and the heat sensation associated with alcohol consumption may increase exposure to water [40]. For instance, an Australian study reported that there was a higher tendency to see people consuming alcohol alongside rivers on hot days [41].

To date, we have not found any study on the association of RH with drowning mortality. Among prior studies, RH is often treated as a confounding variable in the model fitting [42,43]. In our study, we observed that RH was negatively associated with the risk of drowning in an individual analysis of meteorological factors and negatively contributed to drowning mortality in most subgroups in a combined-effect analysis. The underlying mechanism of this finding is unclear and requires further in-depth studies in the future.

In terms of DS, existing studies mainly focus on sunlight exposure and skin cancer or suicide [44-46]. The combined effect analysis in our study shows that DS was the most important weather factor in increasing drowning mortality in all subgroups. Possible explanations for this are that (1) longer daylight hours may be associated with higher temperatures, thereby increasing opportunities for water activities, and (2) light has the neurobiological effect of interacting with the brain serotonin system and may influence serotonin-related behavior [46]. Finally, when daylight hours are short, people are less likely to be active outside and are likely to reduce their contact with bodies of water, which in turn may lead to a decreased incidence of drowning.

We further observed that rainfall was a negative factor for unintentional drowning risk in the combined-effect analysis. Previous studies have shown a negative correlation between rainfall and physical activity and a positive association with sedentary time during weekdays [47]. Therefore, rainfall might make it more difficult for people to work outdoors, which in turn may reduce the chance of contact with bodies of water. In contrast, Murray and Carter [48] showed that high rainfall (180.55 mm to 202.25 mm) was associated with increased drowning deaths in Fiji. The rainfall amount in this study was less than 35 mm, which is more likely to reduce people’s willingness to go outside; thus, we observed a reduced risk of drowning. In addition, the deaths in this study did not include flood-related drownings or disaster drownings.

In the stratified analysis, we further observed that males exhibited higher risk than females when exposed to meteorological mixed factors, which is consistent with previous studies. For example, the risk of drowning death is 1.67 times higher in men than women on hot days [14]. Compared to females, males are more likely to work in hot outdoor environments, resulting in potentially greater access to bodies of water and higher risk of drowning. We also observed that for people aged 0 to 24 years, there was no statistically significant association between mixed exposure to the 4 meteorological factors and drowning. This is contrary to previous studies. For instance, a study in Shanghai reported that drowning deaths among children aged 0 to 14 years mainly occurred in the hot season, while other age groups showed less seasonality in drowning [49]. Possible reasons for this are that the young population is more likely to be influenced by individual personality traits (eg, an active and strong spirit for exploring outside) and behavioral patterns and less influenced by external weather factors and the environment; on the other hand, children also end up in bodies of water due to unintentional falls and fights. Meteorological exposure mixture had the greatest effect on people aged 25 to 64 years; one possible explanation for this finding was that adults were more likely to consume alcohol in hot weather, and drinking alcohol increases exposure to water [50]. Moreover, this age group is the main workforce, with a high chance of occupational exposure to bodies of water, which may increase drowning deaths [51].

Several previous studies have estimated the risk of drowning associated with exposure to a single meteorological factor. However, people are exposed to a variety of meteorological factors simultaneously, so it is important to use an exposure mixture approach to measure the combined effects of weather factors on human health. In this study, we estimated the combined effects of 4 weather factors using a quantile g-computation model. This model helps design potential public health interventions for specific sources of exposure [19].

Multiple sectors in the government, such as education departments, health departments, and meteorological departments, should collaborate to develop an early warning system taking into account DS as a potentially critical weather factor to reduce the risk of drowning in the high-risk season. In addition, in areas with a high risk of drowning, governments should provide adequate and safe swimming places to reduce the possibility of people going into dangerous waters for recreation.

### Limitations

This study has several limitations that must be noted. First, due to the lack of individual exposure data, we used meteorological data obtained by spatial interpolation as a proxy in each city, and there may be some exposure misclassification bias. Second, although this study is based on 5 provinces, which are to some extent nationally representative, extrapolation of the results needs to be done with caution. Third, drowning is influenced by family factors and socioeconomic factors, which were not included in this study. Finally, we only included unintentional drowning (ICD-10 codes W65-74) in this study, which excludes drowning due to cataclysm (ICD-10 codes X34-X39) and transportation accidents (ICD-10 codes V01-V99). This may not adequately reflect the true number of drowning deaths [52].

### Conclusion

This study shows that mixed exposure to the 4 weather factors was positively associated with drowning mortality, which was primarily driven by DS and MT. This finding implies that it is necessary to incorporate sunshine hours and temperature into early warning systems for drowning in the future.

## Appendix

Multimedia Appendix 1 Preliminary analysis of air pressure and wind speed in a generalized additive model.

Multimedia Appendix 2 Spearman correlations between meteorological factors and PM2.5.

Multimedia Appendix 3 Sensitivity analysis, changing the degrees of freedom in a spline function with mean temperature, relative humidity, sunlight duration, and rainfall in a generalized additive model.

Multimedia Appendix 4 The modification effects of city-level characteristics.

Multimedia Appendix 5 Sensitivity analysis with a quantile g-computation model.

## Abbreviations

ANUSPLIN: Australian National University Splines
DS: duration of sunlight
ER: excess risk
GAM: generalized additive model
ICD-10: International Classification of Diseases, 10th Edition
MT: mean temperature
OR: odds ratio
PM: particulate matter
RH: relative humidity
WHO: World Health Organization

## Data Availability

The data sets generated and/or analyzed during this study are available from the corresponding author on reasonable request.
