# Supplementary figures and images for: The Combined Effects of Short-Term Exposure to Multiple Meteorological Factors on Unintentional Drowning Mortality: Large Case-Crossover Study
Source: JMIR Public Health Surveill. 2023 Jul 20;9:e46792. doi: 10.2196/46792 (PMC10401198; doi:10.2196/46792)

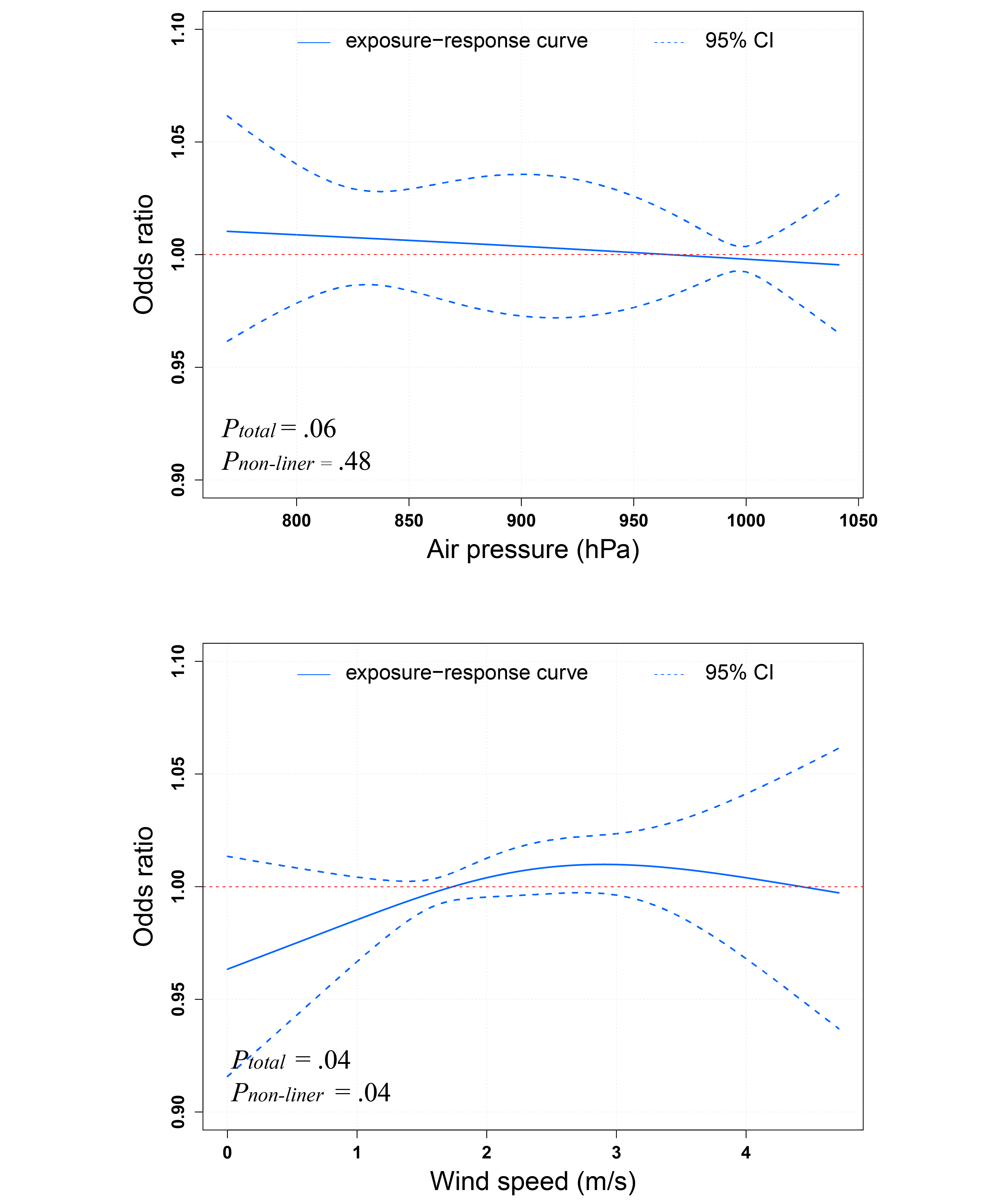

Supplement: Multimedia Appendix 1 [file publichealth_v9i1e46792_app1.png]

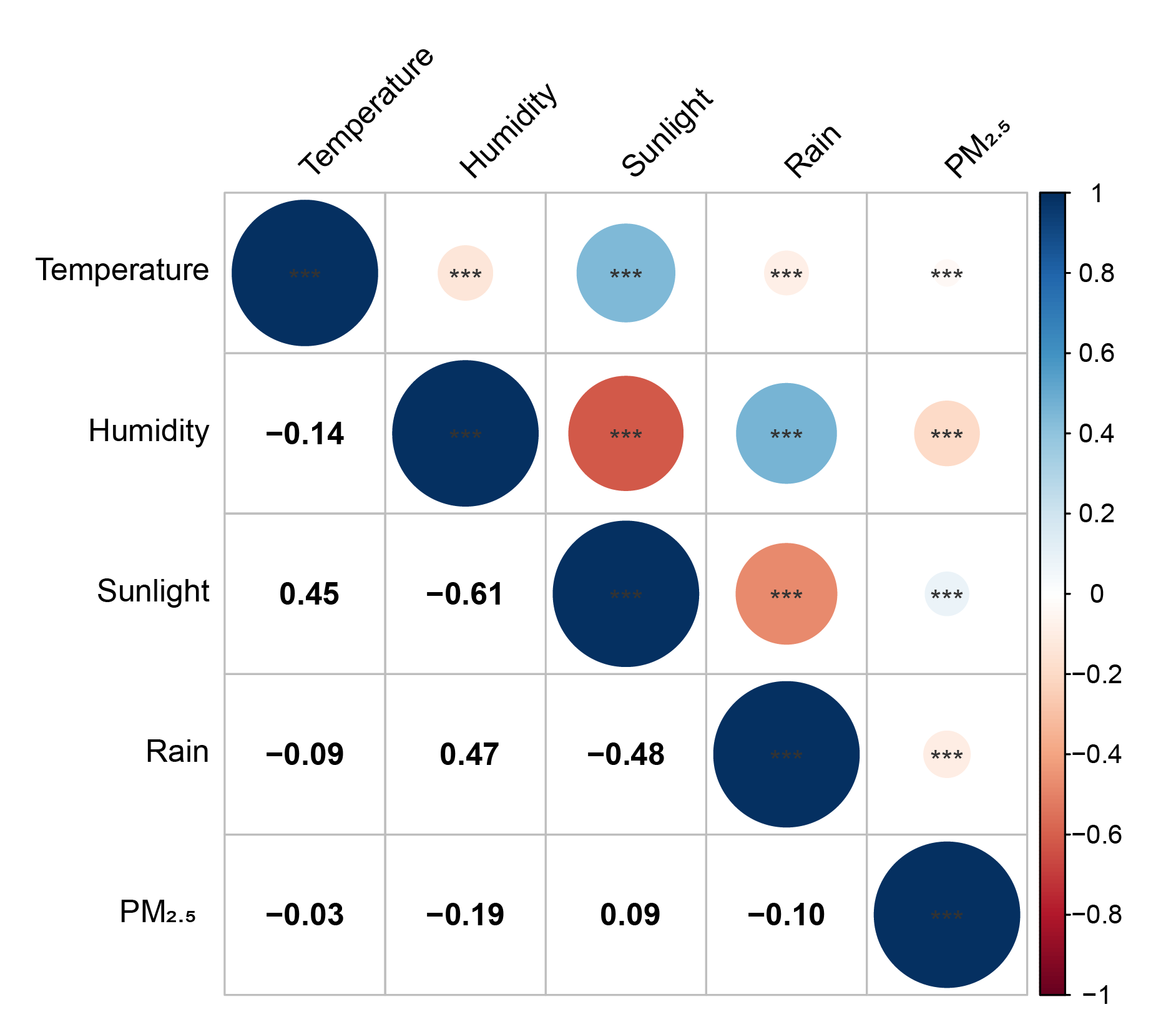

Supplement: Multimedia Appendix 2 [file publichealth_v9i1e46792_app2.png]

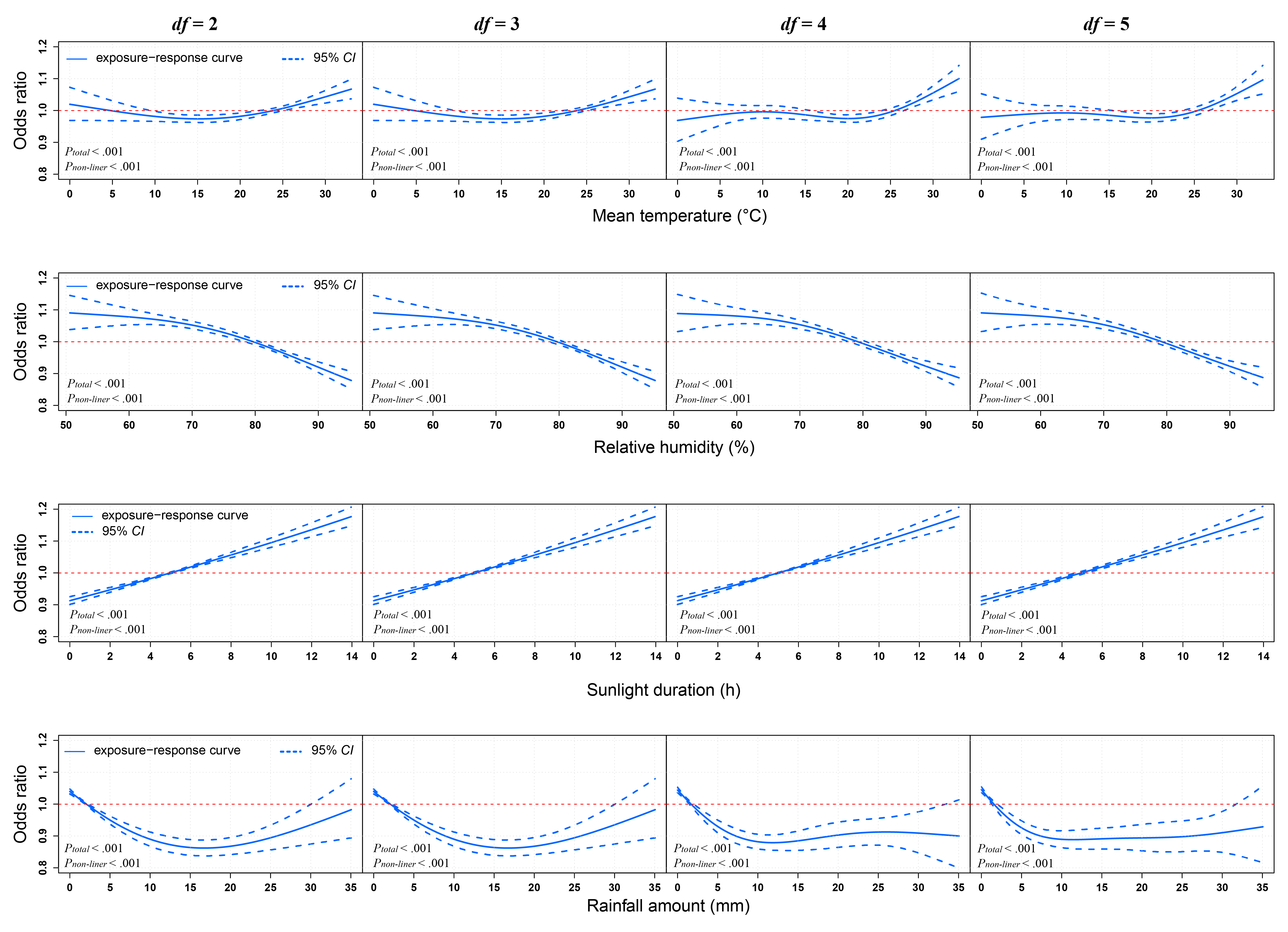

Supplement: Multimedia Appendix 3 [file publichealth_v9i1e46792_app3.png]

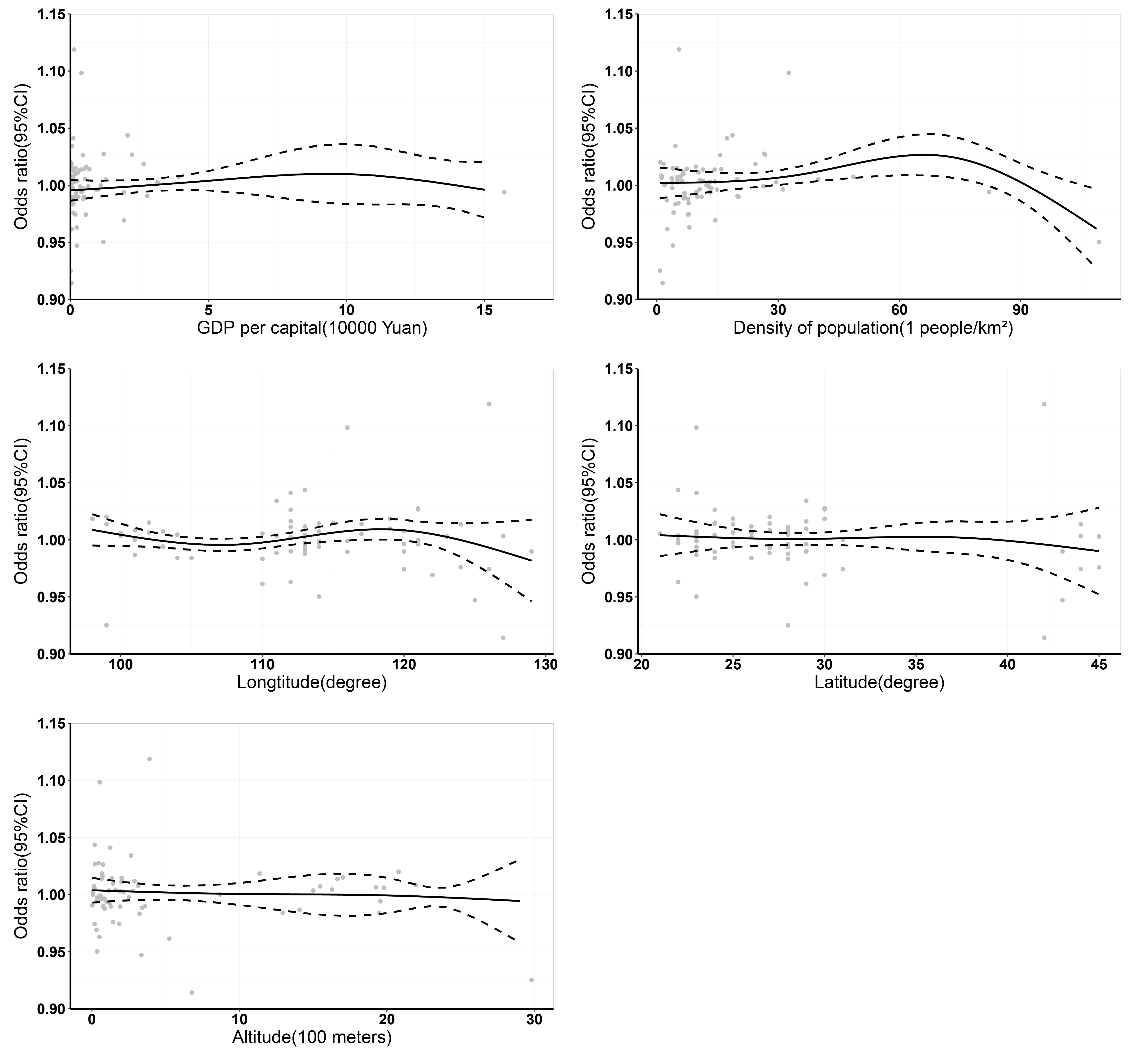

Supplement: Multimedia Appendix 4 [file publichealth_v9i1e46792_app4.png]
