# Supplementary material for: The Combined Effects of Short-Term Exposure to Multiple Meteorological Factors on Unintentional Drowning Mortality: Large Case-Crossover Study
Source: JMIR Public Health Surveill. 2023 Jul 20;9:e46792. doi: 10.2196/46792 (PMC10401198; doi:10.2196/46792)
Supplement: Multimedia Appendix 5 [file publichealth_v9i1e46792_app5.docx]

Sensitivity analysis in quantile g-computation model.

| **Sensitivity analysis** | **Excess Risk [%, (95%CI)]** |
| --- | --- |
| Original model | 3.05(0.37,5.81) |
| Model 1 (remove confounding factor PM_2.5_) ^a^ | 2.80(0.47,5.17) |
| Model 2 (lag 0-1 day) ^b^ | 3.34(0.80,5.94) |
| Model 3 (lag 0-2 day) ^c^ | 3.73(1.27,6.24) |
| Model 4 (lag 0-3 day) ^d^ | 3.50(0.99,6.07) |

Notes:

^a^ Model 1 removes confounding factors PM_2.5_ for sensitivity analysis.

^b^ Model 2 uses 2-day moving average data (TM, RH, PM_2.5_) for the analysis.

^c^ Model 3 uses 3-day moving average data (TM, RH, PM_2.5_) for the analysis.

^d^ Model 4 was analyzed using 4-day moving average data (TM, RH, PM_2.5_).
